# A retrospective observational study of clinicopathological features of *KRAS*, *NRAS*, *BRAF* and *PIK3CA* mutations in Japanese patients with metastatic colorectal cancer

**DOI:** 10.1186/s12885-015-1276-z

**Published:** 2015-04-11

**Authors:** Akihito Kawazoe, Kohei Shitara, Shota Fukuoka, Yasutoshi Kuboki, Hideaki Bando, Wataru Okamoto, Takashi Kojima, Nozomu Fuse, Takeharu Yamanaka, Toshihiko Doi, Atsushi Ohtsu, Takayuki Yoshino

**Affiliations:** 1 Department of Gastroenterology and Gastrointestinal Oncology, National Cancer Center Hospital East, 6-5-1 Kashiwanoha, Kashiwa, Chiba 277-8577 Japan; 2 Exploratory Oncology Research & Clinical Trial Center, National Cancer Center, Chiba, Japan; 3 Department of Biostatistics, Yokohama City University, Kanagawa, Japan

**Keywords:** Colorectal cancer, *KRAS*, *NRAS*, *BRAF*, *PIK3CA*, Epidermal growth factor

## Abstract

**Background:**

The mutation in *KRAS* exon 2 is a validated biomarker of resistance to anti-epidermal growth factor receptor (EGFR) therapy in metastatic colorectal cancer (mCRC). Several reports have confirmed associations of other *RAS* mutations with resistance to anti-EGFR therapy. However, the impact of *BRAF* and *PIK3CA* mutations on the efficacy of anti-EGFR therapy remains controversial. Little is known about the frequencies and clinicopathological features of these mutations, as well as the therapeutic effects of anti-EGFR therapy in mCRC patients with these mutations, especially in the Asian population.

**Methods:**

In this retrospective observational study, frequencies and clinicopathological features of *KRAS*, *NRAS*, *BRAF* and *PIK3CA* mutations were evaluated in patients with mCRC. Among patients treated with anti-EGFR therapy, objective response, progression-free survival (PFS), and overall survival (OS) were evaluated according to gene status.

**Results:**

Among 264 patients, mutations in *KRAS* exon 2, *KRAS* exons 3 or 4, *NRAS*, *BRAF* and *PIK3CA* were detected in 34.1%, 3.8%, 4.2%, 5.4% and 6.4%, respectively. Thus, a total of 12.1% of patients without *KRAS* exon 2 mutations had other *RAS* mutations. Primary rectal tumors tended to be more frequently observed in *RAS* mutant tumors. *BRAF* mutations were more frequently observed with right-sided colon, poorly differentiated or mucinous adenocarcinoma, and peritoneal metastasis. Among the 66 patients with *KRAS* exon 2 wild-type tumors treated with anti-EGFR agents, PFS (5.8 vs. 2.2 months) and OS (17.7 vs. 5.2 months) were significantly better in patients with all wild-type tumors (n = 56) than in those with any of the mutations (n = 10). The response rate also tended to be better with all wild-type tumors (26.8 vs. 0%).

**Conclusion:**

Other *RAS* and *BRAF* mutations were observed in *KRAS* exon 2 wild-type tumors, which were associated with some clinicopathological features and resistance to anti-EGFR therapy in our patient cohort.

**Electronic supplementary material:**

The online version of this article (doi:10.1186/s12885-015-1276-z) contains supplementary material, which is available to authorized users.

## Background

Colorectal cancer was the third most common cancer in men (746,000 cases, 10.0% of the total) and the second in women (614,000 cases, 9.2% of the total) worldwide in 2012 [1]. Mutations in *KRAS* exon 2 occur in ~35% of all metastatic colorectal cancers (mCRCs) [2,3], and constitutively activate the mitogen-activated protein kinase (MAPK) pathway [4,5]. These mutations are validated biomarkers for resistance to anti-epidermal growth factor receptor (EGFR) therapy in patients with mCRC [6-11]. Although conventional *KRAS* tests are useful to exclude patients without benefit from anti-EGFR therapy, response rates and disease control rates to anti-EGFR antibody monotherapy among patients with *KRAS* exon 2 wild-type tumors are only 13–17% and 51%, respectively [6,7]. Therefore, more accurate patient selection requires identification of other predictive factors to improve the risk–benefit profile of anti-EGFR therapy.

Until recently, there have been no validated biomarkers other than *KRAS* exon 2 mutations. Recently, several reports have shown that other *KRAS* (exons 3 or 4) and *NRAS* mutations (exons 2– 4) occur in ~20% of mCRC patients with *KRAS* exon 2 wild-type tumors, which are associated with resistance to anti-EGFR therapy for mCRC [12-18].

*BRAF* mutations were detected in 5–10% of patients with mCRC with V600E as a hot spot. *BRAF* is a downstream molecule of *KRAS* and the clinical data suggest that *BRAF* V600E mutations are associated with poor prognosis in patients with mCRC [11,12,19-24]. However, the relationship between *BRAF* mutations and the efficacy of anti-EGFR therapy remains controversial [19-22]. Besides the KRAS–BRAF pathway, the other major downstream signaling pathway activated by EGFR is the PI3K–AKT signaling pathway. *PIK3CA* mutations, most of which were in exons 9 and 20, were detected in 10–15% of patients with mCRC. According to a European Consortium report [19], *PIK3CA* mutations in exon 20 but not in exon 9 were associated with resistance to anti-EGFR therapy for mCRC. However, in other studies, no clear correlation between *PIK3CA* mutations and the efficacy of anti-EGFR therapy has been observed [21,22]. Meanwhile, targeting agents for these mutations are under development.

We previously reported that a multi-gene cancer panel with Luminex technology (GENOSEARCH Mu-PACK, MBL, Japan) is useful for detection of 36 mutations in *KRAS* exons 3 or 4, *NRAS*, *BRAF* and *PIK3CA* in a single reaction using 50-ng template DNA from formalin-fixed, paraffin-embedded (FFPE) specimens [25]. Importantly, the analysis of 82 samples was fully concordant with conventional direct sequencing. However, information about the frequencies and clinicopathological features of these mutations in clinical practice, including the relationship between mutation status and the efficacy of anti-EGFR therapy, especially among Asian populations, is still limited.

In the present study, we evaluated the frequencies andclinicopathological features of *KRAS*, *NRAS*, *BRAF* and *PIK3CA* mutations in Japanese mCRC patients, and assessed their corresponding effects on the efficacy of anti-EGFR therapy.

## Methods

### Patients

We have conducted a retrospective observational study in our institution to evaluate the frequencies and clinicopathological features of *KRAS*, *NRAS*, *BRAF* and *PIK3CA* mutations in Japanese mCRC patients. Principal inclusion criteria were as follows: histologically confirmed adenocarcinoma of the colon or rectum; and presence of unresectable metastatic disease.

Between January 2013 and June 2014, we analyzed 264 patients with mCRC who met the inclusion criteria. The study was conducted with the approval of the National Cancer Center Institutional Review Board. Written informed consent was obtained from as many patients as possible. For the deceased patients and their relatives, we also disclosed the study design at the website of National Cancer Center and gave them the opportunity to express their wills in accordance with the Epidemiological Study Guideline of Ministry of Health, Labour and Welfare in Japan.

### Molecular profiling and data analysis

Genomic DNA was extracted from FFPE cancer specimens (239 primary tumors and 25 metastases). A total of 36 mutations were analyzed using Luminex (xMAP) technology (GENOSEARCH Mu-PACK, MBL), including: *KRAS* codon 61 (Q61K, Q61E, Q61L, Q61P, Q61R and Q61H); *KRAS* codon 146 (A146T, A146S, A146P, A146E, A146V and A146G); *NRAS* codon 12 (G12S, G12C, G12R, G12D, G12V and G12A), codon 13 (G13S, G13C, G13R, G13D, G13V and G13A); codon 61 (Q61K, Q61E, Q61L, Q61P, Q61R and Q61H); *BRAF* codon 600 (V600E); *PIK3CA* exon 9 codon 542 (E542K); codon 545 (E545K); codon 546 (E546K); and exon 20 codon 1047 (H1047R, H1047L). The lower limit of the percentage of mutant allele in the tumor samples accepted by the study was 5%. Initially, 50-ng samples of template DNA were collected from FFPE tissue samples and were amplified using polymerase chain reactions (PCRs) with a biotin-labeled primer. Subsequently, PCR products and fluorescent Luminex beads were bound to oligonucleotide probes that were complementary to wild-type and mutant genes, and were hybridized and labeled with streptavidin–phycoerythrin. Subsequently, the products were processed according to Luminex assays and data were analyzed using UniMAG software (MBL). The procedure took ~4.5 h. The status of *KRAS* exon 2 (codons 12 and 13) was evaluated by amplification using a refractory mutation system–Scorpion assay with 1% sensitivity in a central vendor laboratory.

Patient characteristics, including age, sex, site of primary lesion, histology, site of metastases, and treatment results, were collected from medical records. Sites of primary lesions were divided into right colon, left colon, and rectum. Right-sided tumors were defined as those arising anywhere from the cecum to the transverse colon, and left-sided tumors as those arising anywhere from the splenic flexure to the rectosigmoid junction. The efficacy of anti-EGFR therapy was evaluated according to gene status in patients who met the following inclusion criteria: Eastern Cooperative Oncology Group performance status (ECOG PS) score ≤ 2, *KRAS* exon 2 wild type, at least one prior chemotherapy regimen, treatment with anti-EGFR either as monotherapy or in combination with irinotecan or FOLFIRI (5-FU, L-leucovorin and irinotecan), baseline computed tomography (CT) performed within 28 days of anti-EGFR therapy, initial evaluation of treatment effect via CT scan within 3 months of initial anti-EGFR therapy and adequate hematological, hepatic and renal function.

### Statistical methods

Gene mutation frequencies and associations of *RAS* or *BRAF* mutations with clinicopathological features were estimated in mCRC patients.

Response rate (RR) and disease control rate (DCR; including complete or partial response and stable disease) were evaluated for anti-EGFR therapy according to the Response Evaluation Criteria in Solid Tumors (RECIST; version 1.1). Progression-free survival (PFS) was defined as the time from initial administration of anti-EGFR regimens until the first objective evidence of disease progression or death from any cause. Overall survival (OS) was defined as the time from initial administration of anti-EGFR regimens until death from any cause. For PFS or OS, patients were censored at the time of their last follow-up if they were free of disease progression or alive, respectively. PFS and OS rates were estimated using the Kaplan–Meier method, and differences among the groups according to *KRAS*, *NRAS*, *BRAF* and *PIK3CA* gene status were identified by univariate and multivariate analyses using Cox proportional hazards models and presented as hazard ratios (HRs) with 95% confidence intervals (CIs). Confounders in univariate and multivariate analyses included ECOG PS (0 vs. 1 and 2), numbers of metastatic sites (1 vs. ≥ 2), treatment line of anti-EGFR regimens (2nd vs. 3rd) and types of anti-EGFR regimens (monotherapy vs. combination therapy).

The χ^2^ test, Fisher’s exact test, Mann–Whitney *U* test, or Kruskal–Wallis test was used to compare patient characteristics and treatment response, as appropriate. Statistical analyses were performed using IBM SPSS Statistics version 21 (IBM Corporation, Armonk, NY, USA). All tests were two-sided, and differences were considered significant when *P* was < 0.05.

## Results

### Frequencies of *KRAS*, *NRAS*, *BRAF* and *PIK3CA* mutations in mCRC patients

**Figure 1:**
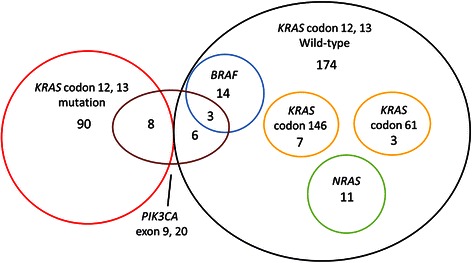
Associations between *KRAS*, *NRAS*, *BRAF* and *PIK3CA* mutations. All mutations in *KRAS* codons 12 and 13, *KRAS* codons 61 and 146, *BRAF* and *NRAS* were mutually exclusive. Mutations in *PIK3CA* exons 9 and 20 overlapped with those in *KRAS* codons 12 and 13 and *BRAF*.

Patient characteristics and frequencies of gene mutations in 264 patients are shown in Tables 1 and 2, respectively. One hundred and thirty-three patients (50.1%) had tumors with no mutation (all wild type). Mutations in *KRAS* codons 12 or 13, *KRAS* codons 61 or 146 and *NRAS* codons 12, 13, or 61 were detected in 90 (34.1%), 10 (3.8%) and 11 (4.2%) patients, respectively. Fourteen (5.4%) patients had *BRAF* codon 600 mutations, and 17 (6.4%) had *PIK3CA* mutations (13 in exon 9 and 4 in exon 20). The genotypes of all samples using HGVS nomenclature are shown in Additional file 1. All mutations were mutually exclusive except for those in *PIK3CA*, and 21 (12.1%) patients without *KRAS* mutations in exon 2 had other *RAS* mutations in either *KRAS* exons 3 or 4 or *NRAS* exons 2 or 3 (Figure 1).

**Table 1:** **Patient characteristics and clinicopathological features according to** ***RAS*** **mutations**

Characteristics		All patients	All*RAS*WT	*KRAS*exon2 MT	Any other*RAS*MT^a^	*P*value
		(*n*= 264, %)	(*n*= 153, %)	(*n*= 90, %)	(*n*= 21, %)	
Age	Median (range)	64 (32–86)	64 (32–82)	64 (38–82)	68 (48–86)	0.32*
Gender	Male	166 (62.9)	94 (61.4)	56 (62.2)	16 (76.2)	0.41**
Primary lesion	Right-sided colon	53 (20.1)	29 (19.0)	21 (23.3)	3 (14.3)	0.08**
	Left-sided colon	70 (26.5)	50 (32.7)	15 (16.7)	5 (23.8)	
	Rectum	141 (53.4)	74 (48.3)	54 (60.0)	13 (61.9)	
Histology	Well, mod	240 (90.9)	133 (86.9)	88 (97.8)	19 (90.5)	0.17**
	Por, muc	24 (9.1)	20 (13.1)	2 (2.2)	2 (9.5)	
Site of metastasis	Liver	137 (51.2)	73 (47.7)	49 (54.4)	15 (71.4)	0.10**
	Lung	100 (37.9)	55 (35.9)	40 (44.4)	5 (23.8)	0.16**
	Lymph node	150 (56.8)	87 (56.9)	49 (54.4)	14 (66.7)	0.59**
	Peritoneum	52 (19.7)	33 (21.6)	15 (16.7)	4 (19.0)	0.64**

*Kruskal–Wallis test; **χ^2^ or Fisher exact test. ^a^any mutations in *KRAS* codons 61 or 146 or *NRAS*. mod: moderately differentiated; MT: mutation type; muc: mucinous carcinoma; por: poorly differentiated adenocarcinoma; well: well-differentiated adenocarcinoma; WT: wild type.

**Table 2:** **Mutation rates of each gene in 264 mCRC patients**

Gene	Wild type	Mutation type	Mutation rate (%)
*KRAS* codon 12,13	174	90	34.1
*KRAS* codon 61, 146	254	10	3.8
*NRAS* codon 12, 13, 61	253	11	4.2
*BRAF* codon 600	250	14	5.4
*PIK3CA* exon 9, 20	247	17	6.4

### Association of *RAS* and *BRAF* mutations with clinicopathological features

We analyzed the correlation between *RAS* or *BRAF* genotypes and the clinicopathological features of mCRC. Primary rectal tumor tended to be more frequently observed in *KRAS* exon 2 and other *RAS* mutant tumors than in *RAS* wild-type tumors (60.0 vs. 61.9 vs. 48.3%, *P =* 0.08) (Table 1), although this was not statistically significant. *BRAF* mutant tumors were more likely to develop in the right colon (57.1 vs. 18.0%, *P =* 0.001), and to have poorly differentiated or mucinous adenocarcinoma (42.9 vs. 7.2%, *P =* 0.001), and peritoneal metastasis (50.0 vs. 18.0%, *P =* 0.009) in comparison with *BRAF* wild-type tumors (Table 3).

**Table 3:** **Clinicopathological features according to** ***BRAF*** **mutations**

Characteristics		*BRAF*WT	*BRAF*MT	*P*value
		(*n*= 250,*%*)	(*n*= 14,*%*)	
Age	Median (range)	64 (32-86)	64 (46-75)	0.53*
Gender	Male	160 (64.0)	6 (42.9)	0.15**
Primary lesion	Right-sided colon	45 (18.0)	8 (57.1)	0.001**
	Left-sided colon	67 (26.8)	3 (21.4)	
	Rectum	138 (55.2)	3 (21.4)	
Histology	Well, mod	232 (92.8)	8 (57.1)	0.001**
	Por, muc	18 (7.2)	6 (42.9)	
Site of metastasis	Liver	131 (52.4)	6 (42.9)	0.58**
	Lung	97 (38.8)	3 (21.4)	0.26**
	Lymph node	143 (57.2)	7 (50.0)	0.59**
	Peritoneum	45 (18.0)	7 (50.0)	0.009

*Mann–Whitney *U* test; **χ^2^ or Fisher exact test.

### Efficacy of anti-EGFR therapies according to gene status

#### Patient characteristics

Between January 2013 and June 2014, 66 patients who met the inclusion criteria were treated with second- and third-line regimens containing anti-EGFR agents. Fifty-six patients had tumors with no mutations (all wild-type tumors) and 10 had tumors with mutation in either *KRAS* codons 61 or 146, *NRAS*, *BRAF*, or *PIK3CA* (any of the mutations). Among the 10 patients with any of the mutations, three had *KRAS* codon 146 mutations, two had *NRAS* mutations, two had *BRAF* mutations, two had *PIK3CA* mutations (1 in exon 9 and 1 in exon 20), and one had *BRAF* and *PIK3CA* exon 9 mutations (Table 4).

**Table 4:** **Characteristics of patients who received anti-EGFR therapy**

Characteristics		All WT^a^	Any MT^b^	*P*value
		(*n*= 56, %)	(*n*= 10, %)	
Age	Median (range)	64 (34–79)	64 (51–74)	0.629*
ECOG PS	0	38 (67.9)	3 (30.0)	0.034**
	1–2	18 (32.1)	7 (70.0)	
Primary lesion	Colon	35 (62.5)	5 (50.0)	0.498**
	Rectum	21 (37.5)	5 (50.0)	
Histology	Well, mod	53 (94.6)	8 (80.0)	0.162**
	Por, muc	3 (5.4)	2 (20.0)	
Number of metastasis	1	14 (25.0)	4 (40.0)	0.442**
	>2	42 (75.0)	6 (60.0)	
Treatment line of anti-EGFR mab	2nd line	27 (48.2)	3 (30.0)	0.327**
	3rd line	29 (51.8)	7 (70.0)	
Treatment	Combination therapy	44 (78.6)	3 (30.0)	0.004**
	Monotherapy	12 (21.4)	7 (70.0)	
Gene mutation	*KRAS* codon 61, 146	-	3	
	*NRAS* codon12, 13, 61	-	2	
	*PIK3CA* exon 9, 20	-	3	
	*BRAF* codon 600	-	3	

*Mann–Whitney *U* test; **χ^2^ or Fisher exact test. ^a^Wild type *KRAS* codons 61, 146, *NRAS*, *BRAF* and *PIK3CA*; ^b^any mutations in *KRAS* codons 61 or 146, *NRAS*, *BRAF* or *PIK3CA.* mab: monoclonal antibody.

Patients with any of the mutations were more likely to have worse PS and to be treated with anti-EGFR monotherapy than combination in comparison with all wild-type tumors. No other significant difference was seen between the two groups (Table 4).

#### Response to treatment

Among patients with all wild-type tumors (*n* = 56), complete response, partial response, stable disease and progressive disease were observed in 0 (0%), 15 (26.8%), 29 (51.8%) and 12 (21.4%) patients, respectively. In contrast, among patients with any of the mutations (*n* = 10), complete response, partial response, stable disease and disease progression were observed in 0 (0%), 0 (0%), 5 (50.0%) and 5 (50.0%) patients, respectively. Thus, RR of patients with all wild-type tumors (*n* = 56) and those with any of the mutations (*n* = 10) were 26.8% and 0% (*P =* 0.101), respectively. Although DCR did not differ significantly between the two groups (78.6 vs. 50.0%, *P =* 0.109), DCR with complete or partial response and stable disease after > 3 months was significantly better in patients with all wild-type tumors than in those with any of the mutations (76.8 vs. 10%, *P =* 0.019).

Among the 10 patients with mutations, three were treated with second-line anti-EGFR-containing regimens and seven were treated with third-line regimens. All three patients treated with second-line anti-EGFR therapy were irinotecan-naïve and had stable disease. Among these, one was treated with irinotecan plus panitumumab and showed stable disease after > 3 months. In contrast, all seven patients treated with third-line anti-EGFR therapy were irinotecan refractory and only two had stable disease at < 3 months.

#### Survival analysis

**Figure 2:**
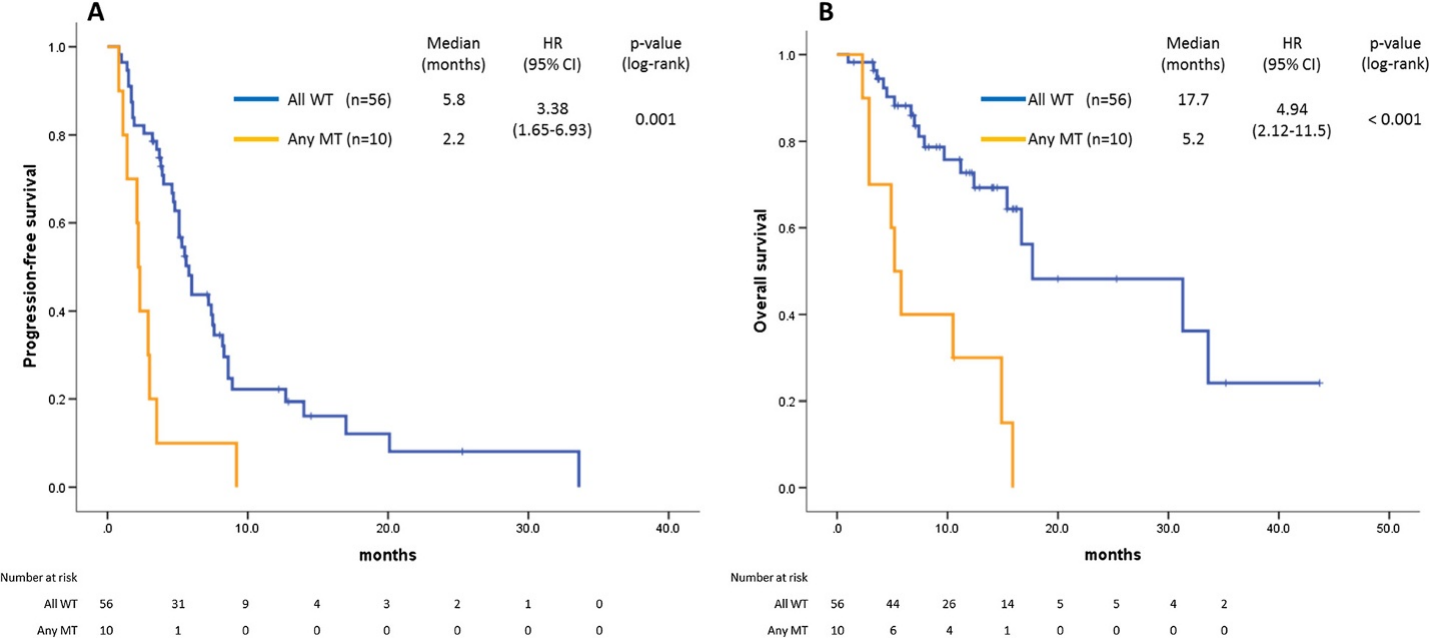
Kaplan–Meier plots of PFS and OS according to *KRAS*, *NRAS*, *BRAF* and *PIK3CA* gene status. **A)** The median PFS was 5.8 months (95% CI, 4.8–6.7) among patients with all wild-type tumors (*n* = 56) and was 2.2 months (1.9–2.5) among those with mutations in *KRAS* codons 61 or 146, *BRAF*, *NRAS* or *PIK3CA* (*n* = 10). Differences in PFS between patients with all wild-type tumors and those with mutations in *KRAS* codons 61 or 146, *BRAF*, *NRAS*, or *PIK3CA* were statistically significant (HR, 3.38; 95% CI, 1.65–6.93; *P* = 0.001). **B)** The median OS was 17.7 months (95% CI, 1.1–34.3) among patients with all wild-type tumors (*n* = 56) and was 5.2 months (3.8–6.6) among those with mutations in *KRAS* codons 61 or 146, *BRAF*, *NRAS* or *PIK3CA* (*n* = 10). Differences in OS values between patients with all wild-type tumors and those with mutations in *KRAS* codons 61 or 146, *BRAF*, *NRAS* or *PIK3CA* were statistically significant (HR, 4.94; 95% CI, 2.12–11.5; *P* < 0.001).

The median PFS of patients with any of the mutations (*n* = 10; 2.2 months; 95% CI, 1.9–2.5 months) was significantly shorter than that of patients with all wild-type tumors (*n* = 56; 5.8 months; 95% CI, 4.8–6.7 months), as verified in both univariate (HR 3.38; 95% CI, 1.65–6.93; *P* = 0.001) and multivariate analyses (HR 2.77; 95% CI, 1.16–6.61; *P* = 0.021) (Figure 2A, Table 5).

**Table 5:** **Univariate and multivariate analyses of PFS and OS**

Variables		No	mPFS (months)	Univariate analysis		Multivariate analysis		mOS (months)	Univariate analysis		Multivariate analysis	
				HR (95% CI)	*P*value	HR (95% CI)	*P*value		HR (95% CI)	*P*value	HR (95% CI)	*P*value
Mutation status	All WT	56	5.8	reference	-	reference	-	17.7	reference	-	reference	-
	Any MT	10	2.2	3.38 (1.65–6.93)	0.001	2.77 (1.16–6.61)	0.021	5.2	4.94 (2.12–11.5)	<0.001	3.38 (1.19–9.58)	0.022
ECOG PS	0	41	6.0	reference	-	reference	-	31.3	reference	-	reference	-
	1-2	25	3.5	2.82 (1.53–5.20)	0.001	1.80 (0.89–3.64)	0.101	9.7	3.41 (1.52–7.69)	0.003	1.62 (0.59–4.42)	0.346
Number of metastasis	1	18	6.0	reference	-	reference	-	15.4	reference	-	reference	-
	>2	48	5.1	1.57 (0.82–3.00)	0.17	1.51 (0.73–3.11)	0.268	16.7	1.70 (0.68–4.29)	0.257	1.60 (0.59–4.30)	0.353
Treatment line of anti-EGFR mab	2nd	30	7.6	reference	-	reference	-	17.7	reference	-	reference	-
	3rd	36	4.0	1.85 (1.06–3.25)	0.032	1.52 (0.83–2.79)	0.174	15.9	1.43 (0.66–3.10)	0.367	0.97 (0.41–2.29)	0.940
Combination therapy	Yes	47	7.4	reference	-	reference	-	31.3	reference	-	reference	-
	No	19	2.6	4.82 (2.49–9.35)	<0.001	2.73 (1.28–5.83)	0.009	10.5	3.31 (1.48–7.41)	0.004	2.03 (0.83–4.96)	0.121

mOS, median overall survival; mPFS, median progression-free survival.

The median OS of patients with any of the mutations (*n* = 10; 5.2 months; 95% CI, 3.8–6.6 months) was significantly shorter than that of patients with all wild-type tumors (*n* = 56; 17.7 months; 95% CI, 1.1–34.3 months), as verified in both univariate (HR 4.94; 95% CI, 2.12–11.5; *P* < 0.001) and multivariate analyses (HR 3.38; 95% CI, 1.19–9.58; *P* = 0.022) (Figure 2B, Table 5).

## Discussion

We elucidated the prevalence of *KRAS*, *NRAS*, *BRAF* and *PIK3CA* mutations in Japanese mCRC patients, and clarified the relationship between gene status and clinicopathological features, including the efficacy of anti-EGFR therapy. To date, clinical evidence about these mutations in mCRC has been based on clinical studies in western countries. The present study is believed to be the first to provide information on frequency and type of *KRAS*, *NRAS*, *BRAF* and *PIK3CA* mutations in Japanese patients with mCRC. In addition, the clinical feasibility of the present novel multiplex kit was demonstrated.

In our patient cohort, the frequency of patients with *KRAS* exon 2 (34.1%) mutant tumors was similar to that in previous studies [2-4]. A total of 12.1% of patients without *KRAS* exon 2 mutations had other *RAS* mutations, which was lower than that in recent studies from western countries, which showed 15–26% of these mutations [12-18]. Another previous study from Japan showed that other *RAS* mutations were detected in seven (12.7%) of 55 samples without *KRAS* exon 2 mutations with 3–13% sensitivity [26], which was similar to our result. Several possible explanations for the relatively lower frequency of other *RAS* mutations in our study compared with western studies might be considered. First, there were some differences in detectable *RAS* mutations by multiplex kit between our study and western studies. In our study, we did not analyze *KRAS* codons 59 and 117 and *NRAS* codons 59, 117 and 146, while these codons were analyzed in most western studies. Although the frequencies of these mutations are considered to be low, it might be one of the causes of the lower frequency in our patient cohort. Second, the sensitivity of *RAS* mutation analysis may vary among studies. In the present study, all mutations were detectable with 5–10% sensitivity. In contrast, Surveyor Scan Kits, BEAMing technology and pyrosequencing were used in pivotal studies, and *RAS* mutations were detected with 1–10% sensitivity [12-18]. A recent multicenter study in Japan, including our institution, showed that other *RAS* mutations were detected in 15% of patients with *KRAS* exon 2 wild type, using a newer multiplex kit (MEBGEN RASKET Kit) [27]. This method detected 48 *RAS* mutations in exon 2 (codons 12 and 13), exon 3 (codons 59 and 61) and exon 4 (codons 117 and 146), with 1–5% sensitivity in a single reaction using 50–100-ng DNA from FFPE tissue without manual dissection. Given these methodological differences, further studies are required to confirm differences in the prevalence of other *RAS* mutations between Asian and western populations. In this study, we detected *BRAF* mutations in 5.4% of patients. The prevalence of *BRAF* mutation might be dependent on the patient population studied. mCRC patients with *BRAF* mutant tumors have a poor prognosis, so the prevalence of *BRAF* mutant populations may decline in pretreated patients compared with chemonaïve patients. The prevalence of *BRAF* mutations in our patient cohort was similar to that of previous studies of pretreated patients with mCRC [11,12,19-24].

We also investigated the clinicopathological features of mCRC patients with respect to *RAS* and *BRAF* mutations. Primary rectal tumor tends to be more frequently observed in *KRAS* exon 2 and other *RAS* mutant tumors rather than *RAS* wild-type tumors, although this was not statistically significant. Previous studies showed that *KRAS* exon 2 mutation was significantly higher in the right colon [28,29], in disagreement with our analysis. No significant differences in other clinicopathological features such as age, sex, primary lesion, histology, and site of metastasis were observed between *KRAS* exon 2 and other *RAS* mutant tumors, which is similar to previous studies [30]. Regardless of these clinicopathological features, it is reported that other gene expression profiles based on The Cancer Genome Atlas appear to be similar in patients with *KRAS* and *NRAS* mutant mCRC, suggesting that treatment selection based on molecular profile is important [30]. In accordance with previous reports [23,24], *BRAF* mutant tumors are more likely to develop in the right colon, and to have poorly differentiated or mucinous adenocarcinoma, and peritoneal metastasis in comparison with *BRAF* wild-type tumors.

In agreement with previous studies [19,25], mutations in *KRAS* exons 3 or 4, *NRAS*, *BRAF* or *PIK3CA* were not associated with clinical benefits from anti-EGFR therapy in the present cohort. On the basis of recent prospective and retrospective randomized trials of anti-EGFR therapy [12-18], the National Comprehensive Cancer Network (NCCN) recommends anti-EGFR therapy for mCRC patients without other *RAS* mutant tumors or *KRAS* exon 2 mutant tumors [31]. The Japanese Society of Medical Oncology (JSMO) also recommends testing for all *RAS* mutations in patients with mCRC before anti-EGFR therapy. In contrast, whether *BRAF* and *PIK3CA* mutations are predictive of the efficacy of anti-EGFR therapy remains controversial [19-22]. Previous trials suggest that intensive combination chemotherapy with FOLFOXIRI (5-FU, L-leucovorin, irinotecan, and oxaliplatin) and bevacizumab might be especially effective for *BRAF* mutant mCRC [32]. Recently, the combination of *BRAF* inhibitors and anti-EGFR monoclonal antibodies, with or without PI3K inhibitors or MEK inhibitors, has shown promising results in phase I trials in patients with *BRAF* mutant CRC [33,34]. Patients with *BRAF* mutant CRC are often refractory to systematic chemotherapy and have poor prognosis, therefore, screening for *BRAF* mutations is important during recruitment of patients for these clinical trials. Accordingly, we conducted a multi-institutional screening (GI-SCREEN) study using the present multiplex kit to elucidate the nationwide prevalence of these targetable mutations.

There were several methodological limitations to the present study. First, not all of the patients in our study period were evaluated for their *RAS* gene status. Thus, the analysis may have been subject to some selection bias. Second, the small sample size and single-center population were other major limitations. Owing to the overall small number of patients with *KRAS* exon 3 or 4, *NRAS*, *BRAF* or *PIK3CA* mutations, we could not evaluate the impact of each gene mutation on the efficacy of anti-EGFR therapy. In addition, our analyses were explorative and hypothesis generating. This issue should be analyzed in a larger cohort.

## Conclusions

Other *RAS* and *BRAF* mutations have been observed in *KRAS* exon 2 wild-type tumors, which were associated with some clinicopathological features and resistance to anti-EGFR therapy in our patient cohort. Importantly, because there are a certain number of mCRC patients with molecular alteration other than *KRAS* exon 2, further refinement of tumor-specific genetic markers is needed to improve the efficacy of anti-EGFR therapy.

## Additional file

Additional file 1:
**The genotypes of all samples using HGVS nomenclature.**

## Abbreviations

CI: Confidence interval
CT: Computed tomography
DCR: Disease control rate
EGFR: Epidermal growth factor receptor
FFPE: Formalin-fixed, paraffin-embedded
mCRC: Metastatic colorectal cancer
OS: Overall survival
PCR: Polymerase chain reaction
PFS: Progression-free survival
RR: Response rate
